# An update on antibody–drug conjugates in urothelial carcinoma: state of the art strategies and what comes next

**DOI:** 10.1007/s00280-022-04459-7

**Published:** 2022-08-11

**Authors:** Alberto D’Angelo, Robert Chapman, Marianna Sirico, Navid Sobhani, Martina Catalano, Enrico Mini, Giandomenico Roviello

**Affiliations:** 1 Department of Biology and Biochemistry, University of Bath, Bath, BA2 7AY UK; 2 Department of Medicine, Princess Alexandra Hospital NHS Foundation Trust, Harlow, CM20 1QX UK; 3 Department of Medical Oncology, IRCCS Istituto Romagnolo per lo Studio dei Tumori (IRST) “Dino Amadori”, Meldola, Italy; 4 Section of Epidemiology and Population Science, Department of Medicine, Baylor College of Medicine, Houston, TX 77030 USA; 5 School of Human Health Sciences, University of Florence, Largo Brambilla 3, 50134 Florence, Italy; 6 Department of Health Sciences, University of Florence, vialePieraccini, 6, 50139 Florence, Italy

**Keywords:** Antibody–drug conjugate, Urothelial cancer, Enfortumab vedotin, HER2, Immunotherapy, Nectin-4, Sacituzumab govitecan, Bladder

## Abstract

In recent years, considerable progress has been made in increasing the knowledge of tumour biology and drug resistance mechanisms in urothelial cancer. Therapeutic strategies have significantly advanced with the introduction of novel approaches such as immune checkpoint inhibitors and Fibroblast Growth Factor Receptor inhibitors. However, despite these novel agents, advanced urothelial cancer is often still progressive in spite of treatment and correlates with a poor prognosis. The introduction of antibody–drug conjugates consisting of a target-specific monoclonal antibody covalently linked to a payload (cytotoxic agent) is a novel and promising therapeutic strategy. In December 2019, the US Food and Drug Administration (FDA) granted accelerated approval to the nectin-4-targeting antibody–drug conjugate, enfortumab vedotin, for the treatment of advanced or metastatic urothelial carcinomas that are refractory to both immune checkpoint inhibitors and platinum-based treatment. Heavily pre-treated urothelial cancer patients reported a significant, 40% response to enfortumab vedotin while other antibody–drug conjugates are currently still under investigation in several clinical trials. We have comprehensively reviewed the available treatment strategies for advanced urothelial carcinoma and outlined the mechanism of action of antibody–drug conjugate agents, their clinical applications, resistance mechanisms and future strategies for urothelial cancer.

## Introduction

The treatment options for advanced urothelial cancer (UC) have been rapidly developing over the last few years. This development began with the approval of anti Fibroblast Growth Factor Receptor (FGFR) and various immune checkpoint inhibitors (ICIs), followed by the Food and Drug Administration (FDA) approving enfortumab vedotin (EV), an antibody–drug conjugate (ADC) for the treatment of advanced urothelial carcinoma in 2019 [1]. Whilst EV is the first ADC to gain FDA approval for the treatment of UC, their use is not novel and are commonly used for the treatment of breast cancer [2, 3] and hematologic malignancies [4, 5]. Given the positive results observed with EV in patients with advanced UC, several clinical trials are underway with the aim of demonstrating the improved efficacy of ADCs and with the ultimate goal of approving the ADC use in earlier lines of therapy. This review will provide an overview of current treatment options for advanced urothelial carcinoma and details of the clinical development of various ADCs being studied for use in this cancer type.

## Urothelial carcinoma

Urothelial cancer is the fourth most common cancer among American males with over 80,000 cases diagnosed in 2020 [6]. UC normally occurs in older patients (7th decade and older). Risk factors for development include a genetic predisposition (such as Lynch syndrome), chemical and environmental exposure (cyclophosphamide, aromatic amines), cigarette smoking and male sex [7]. The vast majority of urothelial cancers arise within the bladder and are found to have not yet invaded the muscle at diagnosis [8]. First-line treatment for non-muscle invasive bladder cancer (NMIBC) (Ta/Tis/T1) is generally surgical via transurethral resection of bladder tumour (TURBT) with additional intravesical therapy using bacillus Calmette-Guerin (BCG) or mitomycin, in order to prevent either disease relapse or regression [9, 10].

Even though a large number of patients benefit from the aforementioned strategy, a fraction of these patients will progress to muscle-invasive bladder carcinoma (MIBC), a locally advanced disease stage that is associated with a high rate of lymph node spread and distant metastasis [11]. In these cases, the gold standard treatment involves neoadjuvant cisplatin-based chemotherapy (although still poorly adopted compared to adjuvant chemotherapy) followed by radical cystectomy, a surgical procedure associated with non-trivial mortality and a significant effect on quality of life [12]. As a result, it is clear that in certain cases there is a significant unmet clinical need and thus an opportunity for future drug development.

## Treatment of advanced muscle-invasive urothelial carcinoma

Systemic treatment is required for patients with advanced or metastatic urothelial cancer (mUC). Until recently, cisplatin-based combination therapies were the only option available. Examples included: methotrexate, vinblastine, adriamycin and cisplatin (MVAC), or gemcitabine and cisplatin (GC). In a phase III trial, both regimens demonstrated a similar response rate of approximately 40–50% and a 5-year survival rate of 10–15%. However, GC regimens reported lower toxicity and are therefore currently the treatment of choice in ongoing trials [13]. Conversely, regimens containing carboplatin (usually gemcitabine plus carboplatin), are the preferred treatment for those deemed cisplatin-ineligible, with similar response rates to GC regimens, but with poorer survival outcomes [14, 15].

Further research in this setting has led to the development of immune checkpoint inhibitors, such as atezolizumab, avelumab, durvalumab, nivolumab and pembrolizumab; all of which are now can be used in patients with UC [16]. Phase III trials recently demonstrated that pembrolizumab treatment resulted in a 3 months benefit in terms of survival when compared to standard of care taxane or vinflunine in platinum-ineligible patients [17, 18]. Notably, in another phase III trial, atezolizumab showed no benefit on overall survival. Nonetheless, both atezolizumab and pembrolizumab were granted FDA approval for their use as first-line agents for platinum-ineligible patients with UC [19].

In addition to immunotherapy options, second-line regimens including antifolates and taxanes represent a valid option for patients who have had disease progression on platinum-based regimens, although these regimens do report poor response rates of approximately 15% [17]. Additionally, a vinflunine agent reported a debatable improvement in survival when compared to the standard of care in a phase II trial, achieving European Medicines Agency but not FDA approval [20].

Moreover, significant treatment advancements have been made for those metastatic UC patients (20%) harbouring mutations in the FGFR pathway. Erdafinitib, an oral FGFR-inhibitor, reported a response rate of 40% in patients with FGFR alterations in phase II single study, leading to accelerated FDA approval for use in patients who have progressed on platinum-based therapy [21, 22]. Despite these developments, a large number of patients still relapse following platinum-based regimens as well as immunotherapy. Consequently, this unmet clinical need led to the generation of antibody–drug conjugates, which have generated a robust interest within the scientific community.

## Antibody–drug conjugates (ADCs)

ADCs are small molecule anticancer agents covalently linked to a monoclonal antibody (mAb). Specific antigens expressed on tumour surfaces are targeted by the mAb, resulting in selective delivery of the anticancer agent to tumour cells [23] (Table 1). Excessive toxicity or poor handling limits some chemotherapeutic drugs from being used as classical chemotherapeutic agents [24]. ADCs can overcome this limitation by selectively delivering cytotoxic agents to tumour targets, reducing toxicity, and increasing efficacy. The early development of ADCs involved the use of murine antibodies conjugated with chemotherapeutic agents such as methotrexate, doxorubicin, and vinblastine, even though these agents have limited selectivity and strong immunogenicity [25]. As the technology has evolved and humanised antibodies developed, these agents have become more effective and specific, leading to improved potency and reduced immunogenicity [26]. Three components make up an ADC; an antibody specific for the target antigen, a linker domain and then the cytotoxic agent.

**Table 1:** Pharmacological and clinical data of ADCs in urothelial carcinoma

ADC	Trial	Antibody target	Target expression in UC	Cytotoxic payload and mechanisms	Chemical linker	References	Phase	Sample size	Outcomes
Enfortumab vedotin	EV-101	Nectin-4 (also PCRL-4)	Nectin-4 is a transmembrane protein ubiquitously expressed in bladder tumours (more than 80%)	MMAE (blocks cell division by blocking microtubule polymerization)	Protease-cleavable	Rosemberg et al. [118]	I	155	mOS:12.3 mPFS: 5.4 ORR: 43%
	EV-201					Rosenberg et al. [119]	II	125 89	mOS: 11.7 mPFS: 5.8 ORR: 54%
	EV-301					Powles et al. [55]	III	608	ORR: 40 vs 17.9% mPFS: 5.5 vs 3.7 mOS:11.7
Sacituzumab govitecan	IMMU-132	Trop-2	Trop 2 is a transmembrane protein widely expressed in UC (the amount depends on disease progression)	SN-38 (an active metabolite of irinotecan; it induces double-stranded DNA breaks and causes cell death)	Acid-labile	Bardia et al. [69]	I/II	45	mOS:16.8 mPFS: 6.8 ORR: 28.9
	TROPHY-U-01					Tagawa et al. [68]	II	113	mOS: 10.9 mPFS: 5.4 ORR: 27%
Sirtratumab vedotin	NA	SLITRK-6	SLITRK-6 blocks microtubules blocks cell division, which is a common process in cancer	MMAE (blocks cell division by blocking microtubule polymerization)	Enzyme- cleavable	Petrylak et al. [71]	I	51	mOS: NA mPFS: 4 ORR:33%
Disitamab edotin	NA	HER-2	HER-2 blocks microtubules blocks cell division, which is a common process in cancer	MMAE (blocks cell division by blocking microtubule polymerization)	Enzyme- cleavable	Sheng et al. [80]	II	43	mOS: 13.9 mPFS: 6.9 ORR: 51%

*MMAE* Monomethyl auristatin E, *HER2* human epidermal growth factor receptor 2, *NA* Not Applicable, *mPFS* median progression-free survival, *mOS* median overall survival, *ORR* objective response rate

### Antibody identification

**Fig. 1:**
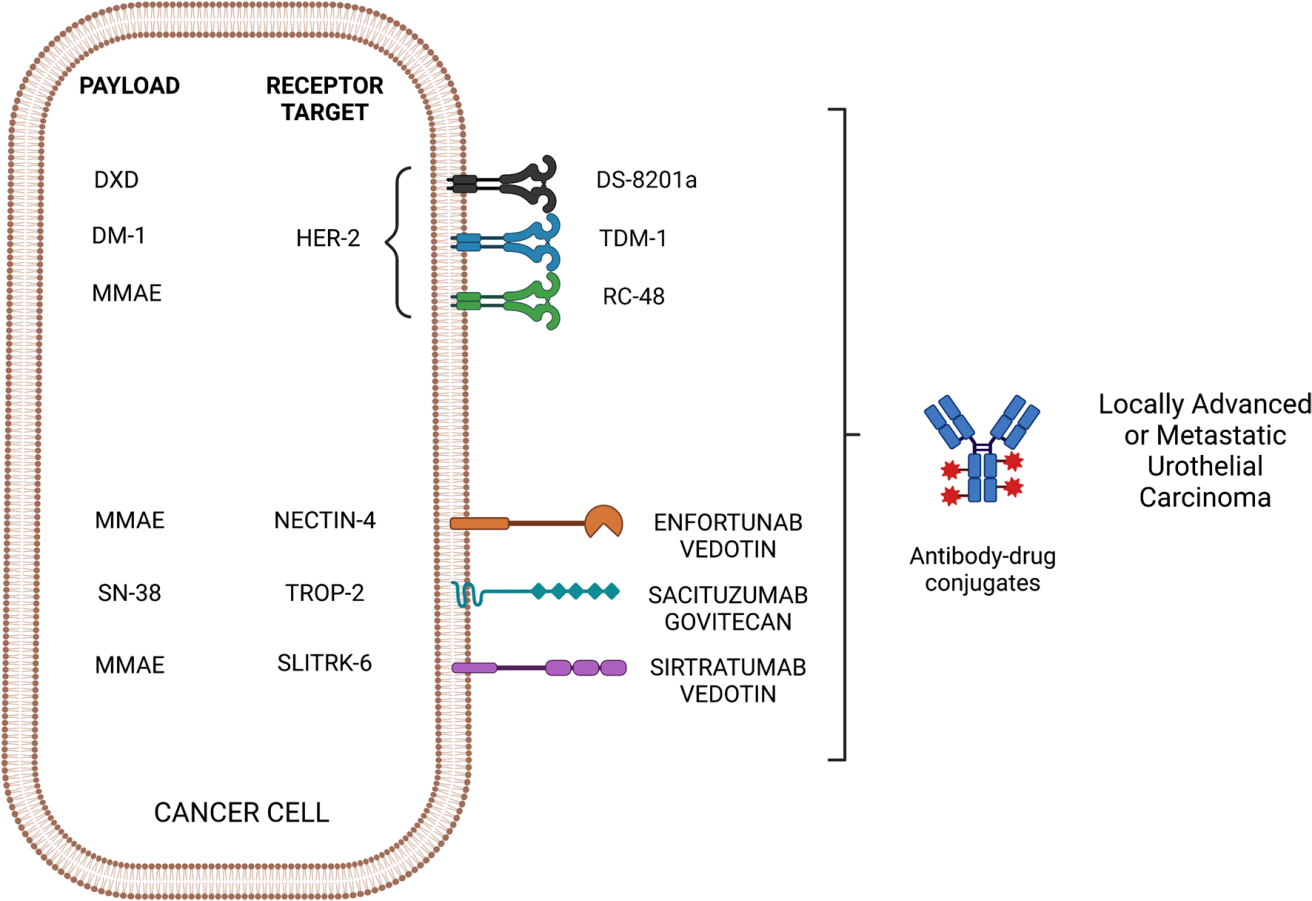
Different types of ADCs tested in urothelial cancer. *DXD *deruxtecan,* DM-1* emtansine, *MMAE *monomethyl auristatin E,* HER2 *human epidermal growth factor receptor 2,* T-DM1 *trastuzumab emtansine,* TROP-2 *Trophoblast cell surface antigen 2,* SLITRK *Slit- and Trk-like protein

ADCs are not required to elicit an immune response after linking with the cytotoxic payload [27]. Presently, immunoglobulin G forms the integral structure and is composed of four subclasses (IgG1, IgG2, IgG3, and IgG4), each of which differ from each other in the structure of the constant domain and hinge regions [28, 29]. Most immunotherapies, including ADCs, utilise IgG1 as it can stimulate immune effector functions (receptor binding, endocytosis and downstream activation of immune pathways). IgG1 also has the advantage of high stability in serum, and low molecular weight and is well distributed in the intra- and extravascular space [26] (Fig. 1).

The mAb should be targeted against an antigen strongly expressed on malignant cells, and absent on non-malignant cells, this is crucial in reducing systemic toxicity and widening the therapeutic window. Examples of such antigens within UC cells include HER2, Nectin-4 and Trop-2. Furthermore, there should be limited antigen cross-reactivity, with strong binding affinity to the target to ensure effective internalisation and stability [30, 31].

### Drug-mAb linker

The linker acts to join the cytotoxic agent to the antibody via the conjugation sites in the antibody heavy chains. Two crucial characteristics must be present for the Linker to be functional. Firstly, the linker must ensure the antibody and cytotoxic agent remain firmly bound, particularly in the plasma during circulation. An unstable linker may lead to premature delivery of the cytotoxic agent into the systemic circulation leading to unwanted toxicity and reduced therapeutic efficacy [32]. Secondly, the linker must be able to deliver the drug once at the tumour site [33].

There are two main sub-classes of linkers; cleavable and non-cleavable. Cleavable linkers rely on factors in the tumour microenvironment to stimulate the breakdown and release of the ADC cytotoxic agent [34]. Mechanisms of linker cleavage are diverse; one such mechanism is driven by glutathione (highly represented in the cytoplasm compared to the extracellular space), which leads to the release of the cytotoxic agent via breakage of disulphide bonds [35, 36]. A second type of mechanism involves linkers that cleave in environments with an acidic pH, such as hydrazone. This type of linker exploits the acidic pH found in endosomes and lysosomes. However, premature cleavage of these linkers into the circulation may lead to hepatotoxicity such as that described in gemtuzumab ozogamicin [32, 37]. A third type of linker is those that are protease-dependent and are degraded by lysosomal proteases after recognition of a specific peptide sequence. These linkers allow the ADC to be remarkably stable within the plasma and thus avoid a premature release of the cytotoxic agent. Examples of this type of linker include enfortumab vedotin, ADC directed against nectin-4, and sacituzumab govitecan (SG) directed against the human trophoblast cell surface antigen 2 (Trop-2) [38].

Cleavable linkers are less stable than non-cleavable linkers. Non-cleavable linkers rely on the degradation of the complete antibody-linker complex to release the cytotoxic agent. Examples of ADCs with non-cleavable linkers are belantamab mafodotin and trastuzumab emtansine (T-DM1) [39].

### Payloads

The cytotoxic agents in ADCs are often referred to as the payload, and these drugs are usually heavily toxic molecules [40]. The antibody acts as the delivery mechanism of this payload to the tumour target. The early ADCs could deliver classical chemotherapeutic agents, such as methotrexate, vinca alkaloids and doxorubicin [41, 42]. However, ADCs delivering these agents did not demonstrate higher efficacy than when these agents were delivered as standard chemotherapeutic agents [43].

ADC payloads can be split into different macro-categories. The most important of which are the agents that destabilise microtubules, such as auristatins and maytansins, which are derived from natural bacteria. Monomethyl auristatin E and F (MMAE, MMAF) are examples of auristatins and are synthetic derivatives of the dolastatin 10 peptide which is isolated from *Dolabella auricularia* [44]. These drugs act by inhibiting the polymerisation of tubulin, resulting in cell cycle arrest and then apoptosis. Maytansins such as DM1 act in a similar fashion and target tubulin via the vinca alkaloid binding site, subsequently leading to a blockade of mitotic replication and then cell cycle arrest and apoptosis [45, 46].

Other types of payload include those that act directly on DNA damage, examples include cuocarmicins and pyrrolobenzodiazepines. These agents can generate DNA double helix damage and act as alkylating agents leading to disruption of transcription, causing DNA double helix breakage and apoptosis [47–49]. Further examples of this type of payload include the camptothecin analogues, such as SN-38, which can inhibit topoisomerase I resulting in DNA damage and breakage.

Additionally, ADC activity relies on a well-balanced drug-to-antibody ratio (DAR). A high DAR can negatively impact pharmacokinetics [50], whereas a low DAR may reduce ADC potency. ADCs with high DARs may show greater efficacy and internalisation, but this may also lead to increased clearance [51–53]. Importantly, ADCs with lower immunogenicity have a key advantage in that these are less likely to lead to the development of anti-drug antibodies, the presence of which can suppress drug efficacy [54].

## Antitumour activity in urothelial carcinoma

### Enfortumab vedotin

Enfortumab vedotin is a novel ADC composed of a fully human antibody, targeting Nectin-4 and the potent microtubule-disrupting agent monomethyl auristatin E [55]. Nectin-4 is a junction protein implicated in cell–cell adhesion [56]; it is involved in a variety of biological processes such as tumour-cell growth, proliferation, immune modulation and viral entry [57]. In a previous study, it has been found that Nectin-4 mRNA, a poliovirus receptor-related protein-4 (PVRL4), is highly expressed in cancer cells, especially in bladder cancer (BC) [58] and that such aberrant expression is associated with cancer progression and poor prognosis. Due to its central role in tumorigenesis and lymphangiogenesis, it has emerged as a potential biomarker and promising targeted therapy. In 2020, a phase III trial has investigated the efficacy of EV versus investigator-choice chemotherapy (docetaxel, paclitaxel, and vinflunine) in 608 patients progressing after platinum-containing chemotherapy and ICI [55]. At the prespecified interim analysis, the primary endpoint was met with longer overall survival (OS) in the enfortumab vedotin group than in the chemotherapy group [median OS, 12.8 vs. 8.9 months; hazard ratio (HR) for death, 0.70; 95% confidence interval (CI), 0.56–0.89; *p* = 0.001). After these results, enfortumab vedotin was granted EMA and FDA breakthrough therapy approval for the treatment of patients previously treated with platinum-containing chemotherapy and ICI [59].

Moreover, in the first-line setting, EV had a synergistic effect when combined with ICI, based on the results of the ongoing phase Ib/2 EV-103 trial, demonstrating an objective response rate (ORR) of 73%, with 15.6% of complete responses (CR) and median progression-free survival (PFS) of 12.3 months in cisplatin-unfit patients [60]. It is noteworthy to mention that EV is correlated with severe cutaneous adverse reactions, including fatal cases of Steven Johnson Syndrome or Toxic Epidermal Necrolysis, especially during the first cycle of treatment but may occur later, as well as hyperglycemia, pneumonitis, peripheral neuropathy, ocular disorders, infusion-site extravasation, and embryofetal toxicity [55, 61].

### Sacituzumab govitecan

Sacituzumab govitecan is a humanized anti-Trop2 monoclonal IgG1k coupled to the cytotoxic payload, SN-38, the active metabolite of irinotecan and a topoisomerase I inhibitor [62] via a cleavable linker [63, 64]. Trop-2 is a 40-kDa transmembrane glycoprotein that was first discovered in human trophoblast and choriocarcinoma cell line [65]. Due to its short intracytoplasmic tail, it is correlated with several pathways regulating cellular functions such as cell–cell adhesion, cell proliferation and mobility [65, 66]. Moreover, a high Trop-2 expression has been found in different cancers including urothelial cancer where it is associated with aggressive progression and poor survival outcome [67]. The TROPHY-U-01 study is an open-label, single-arm phase II study designed to confirm the SG antitumor activity in patients with metastatic UC who progressed after prior platinum-based and checkpoint inhibitor-based therapies [68].

Among the 113 patients who received SG, central review evidenced an ORR of 27% with an mPFS and mOS of 5.4 months and 10.9 months, respectively; thus confirming the results from the prior phase I/II study showing that SG has significant anticancer activity in heavily pretreated patients [69]. Regarding the adverse events (AEs), it is worth mentioning that SG is generally well tolerated and the observed grade 3 or greater AEs were neutropenia (35%) followed by leukopenia (18%), anaemia (14%), diarrhoea (10%), and febrile neutropenia (10%). Based on this preliminary data, SG received accelerated approval in heavily pretreated patients with mUC who had progressed on platinum and ICIs.

### Sirtratumab vedotin (ASG15-ME)

Sirtratumab vedotin is an ADC composed of a SLITRK6-specific human gamma 2 antibody (Igγ2) conjugated to a small molecule microtubule disrupting agent, monomethyl auristatin E (MMAE) via a protease-cleavable linker [70]. It enables the release of this MMAE to tumours expressing SLITRK6 [71]. This protein belongs to a neuronal transmembrane protein family regulating the growth and survival of neuronal cells in the inner ear that transmit auditory signals. Therefore, mutations in this gene lead to myopia and progressive auditory neuropathy in humans and mice [72, 73]. Several immunohistochemical studies have demonstrated that SLITRK6 is expressed in a variety of epithelial tumours, including lung cancer, glioblastoma and breast cancer, and that it is moderately negatively correlated with tumour malignancy [74].

The first study that reported data on SV anti-tumour activity was a phase I study that included 51 metastatic urothelial cancer patients. SLITRK6 expression was evaluated by immunohistochemistry and results demonstrated it to be positive in 93% of patients. Among the 42 patients treated with a therapeutic dose (> 0.5 mg per kg), 1 showed CR at 39 weeks and 13 had a partial response (PR), resulting in an ORR of 33%. The median duration of response (DOR) and mPFS were 15 and 16 weeks, respectively. SV was generally well tolerated; fatigue was the most common grade 3 or higher AEs, evaluated in 44% of patients [71]. Ten patients experienced reversible ocular toxicities with one grade 3 toxicity. Despite these results, no current ongoing trials are evaluating the SV efficacy in UC metastatic setting.

### Human epidermal growth factor receptor 2 (HER2)—ADCs in bladder cancer

HER2 has a firmly established oncogenic potential in both preclinical and clinical settings, especially in breast cancer [75]. When overexpressed, it leads to the autophosphorylation of tyrosine residues within the cytoplasmic domain of the heterodimer and triggers a complex pathway, resulting in a strong pro-tumorigenic signalling cascade [76]. Recently, various ADCs targeting HER2-positive BC have been investigated, leading to a significant improvement in survival outcomes [77]. Beyond breast and gastric cancer, urothelial carcinoma is the third most prevalent cancer with HER2 overexpression, showing potential utility for HER2-targeting therapy in mUC. Notably, it has been shown that HER2 overexpression was observed in 9.2–12.4% of invasive bladder carcinoma, with 5.1% of those demonstrating a HER2 gene amplification [78]. In addition, Fleischmann et al*.* demonstrated that HER2 amplification was significantly more frequent in lymph node metastases (15.3%) than in matched primary bladder cancers as well as being more apparent in the luminal than in the basal subtypes [79]. Moreover, previous studies demonstrated that in bladder cancer, HER2 overexpression strongly correlated with tumour progression and poor prognosis and, unlike BC, HER2 genomic amplification is not a common mechanism [80, 81]. While in BC the role of HER2-targeting agents has been well defined in both metastatic and adjuvant settings, the efficacy of HER2-targeting agents in bladder carcinomas still remains a challenge.

*Trastuzumab emtansine (TDM-1).*T-DM1 is a HER2-targeted antibody–drug conjugate, combining a monoclonal antibody with an anticancer drug called emtansine, a microtubule inhibitor [82].

Although T-DM1 showed promising antitumor effects in preclinical models of HER2 overexpressing bladder cancers [83], the multi-histology phase II, basket trial of TDM-1 in patients with HER2 amplified cancers failed to demonstrate a significant activity of this drug in patients with mUC..

*Trastuzumab deruxtecan* (T-DXd). T-DXd (DS-8201) is an antibody–drug conjugate that is composed of a humanized monoclonal antibody specifically targeting HER2 linked to potent topoisomerase I inhibitor as the cytotoxic drug (payload) [3]. In the DESTINY-Breast01 trial, DS-8201 showed durable antitumor activity in a pretreated patient population with HER2-positive metastatic breast cancer [3]. Moreover, DS8201 have demonstrated a satisfactory efficacy in patients with metastatic BC HER2 low-expressing [3, 84].

*Disitamab vedotin.*Disitamab vedotin, previously known as RC-48, is a novel ADC consisting of a humanized monoclonal antibody directed against HER-2 conjugated to MMAE via a cleavable linker [85]. In 2021 Shent et al*.,* in a phase II study, evaluated the efficacy and safety of RC-48 in 43 patients with HER2 + (IHC 3 + and 2 +) locally advanced or metastatic UC refractory to standard therapies. They demonstrated a promising efficacy of RC-48 observing an ORR of 51%, an mPFS and mOS of 6.9 and 13.9 months, respectively, with a manageable safety profile [80]. This trial observed a higher ORR compared to historic response rates of currently available ICIs in the second-line setting. Indeed, another phase II trial, enrolling 100 patients, is underway to evaluate whether RC-48 works to treat HER2 expressing urothelial cancer (NCT04879329).

## Mechanism of resistance

Little is currently known about potential resistance mechanisms against ADC treatment in UC. Further investigation is needed to shed light on drug-intrinsic mechanisms and streamline the identification of predictive biomarkers of drug efficacy. Preliminary results link ADC resistance to various biochemical mechanisms including alteration of the cell cycle, loss of payload efficacy, alteration of vesicle pathways and prevention of antibody attachment and loss of target antigen [16, 33].

### Impairment of cell-cycle

It is well established that the cell cycle plays a pivotal role in generating novel resistance mechanisms and a recent study showed that the expression of cyclin B is significantly increased in TDM-1-resistant cells [86]. Furthermore, modifications of the apoptosis pathway might interfere with the efficacy of ADCs. There is evidence of overexpression and mutation of BCL-X and BCL-2, plus impairment of protein regulation of BAX and BAK pathways in patients treated with Gemtuzumab ozogamicin [87].

### Inhibition of payload efficacy

A common mechanism of resistance has been shown to arise following mutations occurring in the molecular target of the payload. For example, the decreased success of SG treatment might be due to resistance mutations in topoisomerase-1 [88]. In addition, ATP-binding cassette (ABC) transporters are deemed to be a frequent mechanism of chemotherapy resistance, acting to increase drug discharge from the cell microenvironment [89]. Several ADC payloads are targeted against ABC efflux transporters, thus conferring resistance to ADC treatment [90, 91]. Myatansinoids and auristatin analogues have been previously reported to be substrates for ABC transporters including multidrug resistance-1 (MDR-1) in preclinical data. Exposing the cell to these agents can result in the overexpression of MDR-1 efflux transporters [92].

### Impairment of vesicle pathways

There is preclinical evidence of decreased treatment sensitivity resulting from the internalization of TDM-1 into caveolin-1-coated vesicles [93]. Although the antibody internalisation into the cell (by endocytosis) is required to promote ADC efficacy, this process might curb payload efficacy. It has been shown that the internalization process might take place by means of clathrin-caveolin-independent, clathrin-mediated, and caveolin-mediated endocytosis mechanisms [94].

### Loss of target antigen

Loss or reduction of target tumour antigen can occur due to a multitude of reasons. Examples include; gene mutation resulting in antigen concealment to the immune system or downregulation of target gene expression or clone selection of those tumour cells with lower target antigen expression. These mechanisms are a common hurdle to maximising ADC treatment efficacy [95] as the loss or reduction of cancer target antigen might result in the release of payload or loss of antibody binding. A study carried out on patients with metastatic triple-negative breast cancer (TNBC) showed that the loss of Trop-2 expression was associated with decreased response to SG treatment [88]. The ASCENT trial compounded that finding by demonstrating that metastatic TNBC patients with high Trop-2 expression, treated with SG, reported better outcomes when compared to those with low or absent Trop-2 expression [96]. Comparably, the EMILIA trial (results of which led to TDM-1 approval for HER-2-positive metastatic cancer patients) observed that patients with higher expression of HER-2 mRNA reported better outcomes when compared to those with lower HER-2 mRNA levels [97]. Nonetheless, preliminary research studies are underway to verify the efficacy of bispecific antibodies (those able to target multiple antigens) with the ultimate goal of overcoming this particular resistance mechanism.

### The role of the tumour microenvironment (TME)

Recent evidence showed that the TME plays a pivotal role in regulating tumour progression, metastasis, immune escape, and it is involved in acquired resistance of tumours to various therapies, resulting in reduced treatment efficacy [98, 99]. Several mechanisms within the TME are deemed to lead to drug resistance. For instance, hypoxia and impaired blood supply, which results from the uncontrolled proliferation of tumour, are a cornerstone of TME in all solid tumours [100]. Hypoxia and impaired blood supply result in abnormal angiogenesis, inflammation and desmoplasia, all of which contribute to tumour progression and therapeutic resistance [101]. Additionally, hypoxia promotes decreased pH in the TME which supports multi-drug-resistances strategies including reduced apoptotic rate, increased activity of multidrug transported p-glycoprotein (P-gp/MDR1)(“drug efflux pump”), genetic alterations such as p53 mutations, and decreased concentration of the drug due to “ion trapping”—namely the inability for charged drugs to diffuse through cells [102].

## Future options to overcome resistance and optimise ADC-based therapy

Although three new ADCs have been recently granted approval for the treatment of solid cancers, a major limit to ADC clinical success is resistance to these drugs. Nonetheless, ADC modular structure and the biochemical improvements will allow soon the development of new agents capable of overcoming resistance. Indeed, ADCs engineering has recently introduced new payloads, linkers and the development of a novel generation of ADCs with an increased drug-to-antibody ratio (DAR) and solid bystander effects [103]. The use of novel cleavable linkers in association with membrane-permeable payload can improve the efficacy of the bystander effect, enabling ADCs to be active against target-negative cells, namely expanding ADC efficacy to cancer with low target expression or on heterogeneous tumours [36, 104]. Another strategy that can be used is the increase of linker hydrophilicity, which can impair drug resistance as P-gp/MDR1 binds to hydrophobic compounds more efficiently than hydrophilic compounds [105]. Other studies have attempted to improve the stability of ADCs in plasma by altering the composition of linkers, focusing on replacing the most susceptible to degradation linker components with more stable substitutes [106].

Additional strategies have recently been investigated to expand the group of patients who might benefit from the newer generation of ADCs. Noticeably, new potential targets including proteins expressed by cancer stem cells (CSC), such as PTK7, ephrin-A4, 5T4 and in the TME, such as CD205, CD25, B7-H3, are under investigation with some of these that already reached clinical phases of drug development [107, 108]. Bispecific and biparatopic antibodies are also under investigation in preclinical studies. While bispecific antibodies can recognize two different antigens on the same antigen, biparatopic antibodies bind two non-overlapping epitopes of the same antigen. Additionally, a newer thread of research is focusing on smarter vehicles for payloads [109]. Probody drug conjugates are a novel group of ADC prodrugs that can be activated following proteolytic cleavage by TME proteases to minimise on-target/off-tumour toxicity [110].

More importantly, the most promising results are coming from several ongoing trials investigating the efficacy of novel ADCs in combination with several targeted agents such as immune checkpoint inhibitors (ICIs).The advent of ICIs and the more recent introduction of FGFR-targeted therapy have significantly altered the treatment paradigm of advanced urothelial cancer. Additionally, the even more recent introduction of ADCs (and the FDA approval of EV) are at the forefront of urothelial cancer treatment with encouraging preliminary and clinical data. ADCs have a multitude of benefits as treatment options. Firstly, response rates are promising (so far demonstrated to be between 30 and 60%) and comparable to current cisplatin-based first-line regimens. Secondly, response to ADCs has been shown to be durable with manageable toxicity. Finally given the universal expression of urothelial cancer targets, a large fraction of patients would most likely be considered eligible or appropriate for ADC treatment. With that in mind, what does the future hold for ADC treatment in urothelial cancer? It is highly likely that additional ADC agents will be granted approval for advanced urothelial cancer treatment, particularly as interest moves toward studying the outcome of ADC therapy in treatment-naïve patients.

Furthermore, the combination of ICIs and ADCs is a strategy that needs to be considered, as the biochemical effect is potentially synergistic with no overlapping toxicity. For example, ADCs can promote cell death, resulting in cancer antigen release, leading to immune system activation and an increase of antigen-presenting cells [111]. At the same time, ICIs can regulate immunosuppression within the tumour microenvironment by modulating cytokines, enzymes and T immune cells with immunomodulatory functions [112]. Therefore, ADC agents have potential synergistic activity in combination with ICIs. The ability of ADCs to modulate the immune system is under investigation in preclinical studies. Gardai et al. reported that ADCs with an MMAE payload can trigger anti-tumour immunity and stimulate immune cell death by induction of damage-associated molecular patterns (DAMPs) on the cell surface which, in turn, can trigger the immune system [113].

Further research in murine models has shown that tumour shrinkage was increased with a PD1 inhibitor and brentuximab combination treatment, corroborating the potential synergism of the two drug classes [114]. According to this preclinical data, it can be fairly confidently assumed that a combination of ADC and ICI might lead to a stronger anti-tumour response in vivo. Notably, combination treatment with ICIs and ADCs has already been granted regulatory approval for several cancers, and different combinations of these two agents are currently under clinical trial investigation. The EV-103 trial, a multi-arm phase Ib/II study investigating the efficacy of EV alone or in association with chemotherapy and/or pembrolizumab in locally advanced urothelial cancer, has reported a greater benefit in terms of ORR (73%) and mPFS (12.3 months) in the first-line setting when combinations are used [115].

In addition, the EV-304 trial, a randomized phase III open-label study in cisplatin-eligible patients is underway for the investigation of early-setting efficacy in patients treated with either EV plus pembrolizumab or neoadjuvant cisplatin in combination with gemcitabine [116]. Another phase III global study, VOLGA, is testing the efficacy and safety of neoadjuvant treatment with EV plus durvalumab and tremelimumab or EV plus durvalumab in cisplatin-ineligible MIBC [117]. As far as the development of novel ADCs is concerned, different agents including integrin β6, EGFR, B7-H1 and CD25 are being evaluated in early phase basket trials with the ultimate goal of better understanding and overcoming primary and acquired resistance mechanisms as well as limiting toxicity (Table 2).

**Table 2:** Ongoing clinical trials investigating ADCs in urothelial carcinoma

NCT identifier	Trial	Drug	Setting	Phase	Characteristics of the study	Recruitment status
NCT03288545	EV-103	Enfortumab vedotin	Metastatic	I/II	The primary goal of the study is to determine the safety, tolerability, and efficacy of Enfortumab vedotin alone and in combination with pembrolizumab and/or chemotherapy in first or second-line therapy in UC	Recruiting
NCT04223856	EV-302	Enfortumab vedotin + pembrolizumab vs. chemotherapy	Metastatic	III	The goal of this open-label, randomized study is to study the efficacy of Enfortumab vedotin in combination with pembrolizumab vs. chemotherapy alone in previously untreated locally advanced or metastatic UC	Recruiting
NCT04225117	EV-202	Enfortumab vedotin	Metastatic	II	The goal of this open-label, multicenter, multicohort is to evaluate the efficacy of Enfortumab vedotin in subjects with previously treated locally advanced or metastatic malignant solid tumours	Recruiting
NCT04960709	VOLGA	Enfortumab vedotin + durvalumab + /-tremelimumab	Perioperative	III	The aim of this randomized, open-label, multicenter study is to determine the safety and efficacy of durvalumab in combination with tremelimumab and Enfortumab vedotin or durvalumab in combination with Enfortumab vedotin for perioperative treatment in patients ineligible for cisplatin undergoing radical cystectomy for muscle-invasive bladder cancer	Recruiting
NCT04963153	NA	Enfortumab vedotin, Erdafitinib	Perioperative	I	The aim of this non-randomized, open-label, single-group assignment study is to determine the incidence of adverse events and maximum tolerability dose of Enfortumab in 30 urothelial carcinoma patients	Recruiting
NCT05239624	NA	Enfortumab vedotin, pembrolizumab	Metastatic	II	The aim of this non-randomized, open-label, single-group assignment study is to determine the pathological complete response rate of Enfortumab in 23 urothelial carcinoma patients	Not yet recruiting
NCT03924895	KEYNOTE- 905/EV-303	Enfortumab vedotin + pembrolizumab vs. pembrolizumab vs. surgery alone	Perioperative	III	This randomized study aims to evaluate the efficacy of cystectomy with perioperative pembrolizumab and cystectomy with perioperative Enfortumab vedotin and pembrolizumab versus cystectomy alone in cisplatin-ineligible participants with muscle-invasive bladder cancer	Recruiting
NCT04700124	KEYNOTE- B15/EV-304	Enfortumab vedotin + pembrolizumab vs. cisplatin + gemcitabine	Perioperative	III	This randomized, open-label study aims to evaluate the safety ad efficacy of perioperative enfortumab vedotin plus pembrolizumab versus neoadjuvant gemcitabine and cisplatin in cisplatin-eligible participants with muscle-invasive bladder cancer	Recruiting
NCT04527991	TROPiCS-04	Sacituzumab govitecan vs. chemotherapy	Metastatic	III	This randomized open-label study aims to investigate the efficacy of sacituzumab govitecan versus treatment of physician’s choice in subjects with metastatic or locally advanced unresectable UC	Recruiting
NCT03547973	TROPHY-U-01	Sacitizumab govitecan	Metastatic	II	The objective of this open-label study is to investigate the safety and efficacy of sacituzumab govitecan in metastatic UC after the failure of a platinum-based regimen or anti-PD-1/ PD-L1 based immunotherapy	Recruiting
NCT04724018	NA	Sacitizumab govitecan, Enfortumab vedotin	Metastatic	I	The aim of this non-randomized, open-label, single-group assignment study is to determine the maximum tolerability dose and dose-limiting toxicity of sacitizumab govitecan and Enfortumab vedotin in 24 urothelial carcinoma patients	Recruiting
NCT05226117	NA	Sacituzumab govitecan	Metastatic	II	The aim of this non-randomized, open-label, single-group assignment study is to determine the pathological complete response rate of sacituzumab govitecan in 56 urothelial carcinoma patients	Recruiting
NCT04482309	DESTINY-PanTumor02	Trastuzumab deruxtecan	Metastatic	II	This multicenter, open-label study aims to evaluate the safety and efficacy of trastuzumab deruxtecan (t-dxd, ds-8201a) for the treatment of selected HER2 expressing tumours (DESTINY-PanTumor02)	Recruiting

*NA* not applicable

## Conclusion

Until recently, chemotherapy was the only treatment available for advanced or mUC. However, over recent years, UC treatment has benefited from multiple advances, and now more targeted therapy exists, in the form of immunotherapy. However, the outcome for these patients remains poor in the long term. ADCs are innovative drug agents, which allow conventional cytotoxic therapies to be transformed into highly targeted chemotherapeutics, potentially enabling better outcomes and reduced toxicity. ADCs offer particular promise in UC as we know that multiple tumour-specific antigens are highly expressed. As a result of this specificity and potential efficacy, ADCs offer a renewed hope for those malignancies with limited therapeutic strategies such as locally advanced or metastatic UC. The ongoing drug development within approved clinical trials will elucidate the optimal sequencing or combination of these drugs. The end goal is a personalized approach to the treatment of urothelial cancer, resulting in improved outcomes for patients.
